# Editorial: Case reports in PET imaging

**DOI:** 10.3389/fmed.2022.1087583

**Published:** 2022-12-02

**Authors:** Silvia Taralli, Natale Quartuccio, Gaurav Malviya

**Affiliations:** ^1^Nuclear Medicine Unit, Dipartimento di Diagnostica per Immagini, Radioterapia Oncologica ed Ematologia, Fondazione Policlinico Universitario Agostino Gemelli IRCCS, Rome, Italy; ^2^Nuclear Medicine Unit, Ospedali Riuniti Villa Sofia-Cervello, Palermo, Italy; ^3^Translational Molecular Imaging, Cancer Research UK Beatson Institute, Glasgow, United Kingdom

**Keywords:** cancer, diagnostics, case report, PET imaging, ^18^F-FDG

Advanced hybrid nuclear imaging technology, including Positron Emission Tomography together with Computed Tomography (PET/CT) or Magnetic Resonance Imaging (PET/MRI), allowing the simultaneous collection of anatomical and functional information, has been gaining significant attention in recent years and has spurred clinical research interests. This Research Topic comprises 12 unique case reports that highlight the role of ^18^F-FDG PET imaging (mostly using PET/CT, with PET/MRI used in a number of cases) in addressing correct differential diagnosis of malignant and benign lesions, guiding the clinical management of patients with an uncommon disease, a challenging diagnosis, or an atypical presentation.

Primary cardiac tumors are very rare (0.001–0.03%), with cardiac paraganglioma (CPGL) representing only 1–3% of cardiac tumors. In this Research Topic, a multimodality imaging case report on unusual CPGL is included, demonstrating that the combination of ^18^F-FDG PET/CT and cardiac magnetic resonance (CMR) offers a useful, non-invasive tool for pre-operative detection and staging of CPGL, which may increase prognosis rates and decrease post-operative complications (Huang et al.). Degrauwe et al. (1) recently demonstrated a similar finding, with CMR and ^18^F-FDG PET/CT providing a unique combination for the proper characterization of morphological and metabolic features in such a tumor. ^18^F-FDG PET/CT can also help in challenging cases of paraganglioma arising at uncommon primary sites such as the bladder (Taninokuchi Tomassoni et al.). Although ^68^Ga-DOTANOC is the most suitable PET radiotracer for identifying bladder paragangliomas (BPGs), ^18^F-FDG may still be a valid and more easily accessible alternative. In the case described in this article, dynamic ^18^F-FDG PET/CT was used to reach a correct diagnosis, with BPG showing early ^18^F-FDG uptake and wash-out in the delayed 1-h acquisition phase.

Similarly, pheochromocytoma is another rare neuroendocrine tumor (2). The case presented by Feng et al. is a good clinical example of the usefulness of PET/CT for detecting pathological findings in patients with pheochromocytoma. The patient evaluated had a history of resected bilateral malignant adrenal pheochromocytoma. After dizziness, hypertension, and bilateral iliac pain for 2 months, a PET/CT was performed, detecting nodal and bone ^18^F-FDG-avid metastases, along with an increased uptake in brown fat in the intercostal muscles, consistent with high levels of catecholamine serum secretion.

Another relatively rare disease, reported in a 3-year-old child, is alveolar rhabdomyosarcoma (ARMS) (Zhang et al.), which is characterized by its aggressive behavior, poor prognosis, and unfavorable outcome. ARMS may be present in critical organs and can invade the spinal canal that leads to the central nervous system; nevertheless, clinical symptoms can be variable, depending on the size and site of the retroperitoneal mass and distant metastases (3). In this patient, a post-operative biopsy and FKHR gene rupture confirmed ARMS, while PET imaging helped to reveal retroperitoneal lymphatic metastases, which was not detected by CT imaging or ultrasound. The authors also emphasized that retroperitoneal ARMS requires careful differentiation, potentially being misdiagnosed as neuroblastoma, which is a very common pediatric extra-cranial, solid tumor. The advantage of using ^18^F-FDG PET/CT for pediatric patients lies in the possibility to evaluate disease activity and therefore guide therapy and prognostication, as is the case for Kaposiform hemangioendothelioma (KHE), a rare vascular neoplasm mostly appearing in infancy or early childhood. A 13-month-old girl with KHE presented with limited movement of the lower extremity caused by spinal involvement and a normal platelet count (Qiu et al.). ^18^F-FDG PET/CT allowed documentation of mild hypermetabolism in the lesion, consistent with a low-grade tumor, as subsequently confirmed by KHE bioptical diagnosis. Treatment with 6-month sirolimus monotherapy reduced the retroperitoneal lesion's size, alleviating clinical symptoms.

As the diagnosis of occult cancers with unknown primary sites is very complicated and challenging, a biopsy is essential for a confident diagnosis; nevertheless, PET/CT or PET/MRI imaging can contribute, in most cases, to the planning of personalized treatment, precise staging, and improved risk-estimation. Zeng et al. reported a case of rare retroperitoneal clear-cell carcinoma with an unknown primary focus. Although no obvious abnormality was observed in either kidney, *via* imaging (PET/CT and MRI) or surgical exploration, interestingly, immunohistochemistry results confirmed a renal origin of the carcinoma. PET/CT revealed enormous ^18^F-FDG activity in the retroperitoneal clear-cell carcinoma (as observed in high-grade renal clear-cell carcinomas), likely due to its pathological grade, suggesting that high ^18^F-FDG uptake could be related to low tumor differentiation.

Besides its widespread oncological application, ^18^F-FDG PET/CT also plays a key role in inflammatory and infectious settings, where its ability to quantify disease extent, evaluate treatment response, and identify inflammatory occult sites, which remain undetermined after clinical and/or conventional imaging assessment, are exploited (4). In this regard, Tsai et al. demonstrated the additional diagnostic value of ^18^F-FDG PET imaging for resolving a challenging case of right-sided persistent lower-back pain after an L5-S1 laminectomy, refractory to several different therapies. When initial spinal root MRI imaging was considered inconclusive to support a surgical intervention, a subsequent ^18^F-FDG PET/MRI revealed increased tracer uptake exclusively along the right S1 root, consistent with the clinical presentation; this metabolic finding allowed the final diagnosis and localization of an inflammatory neuropathy, secondary to epidural fibrosis, with sacral root entrapment. This case highlights the diagnostic value of simultaneous PET/MRI imaging for soft-tissue and spinal cord abnormalities, by combining metabolic and anatomic information.

As stated above, since most tumors demonstrate an increased ^18^F-FDG uptake (greater than background tissues and benign lesions), metabolic information provided by ^18^F-FDG PET imaging often plays a crucial role in correctly differentiating malignant and benign diseases. However, the chance of misinterpreting a hypermetabolic benign finding as malignant requires extreme caution from PET readers, particularly in cases of rare diseases or uncommon presentations, which can potentially lead to unnecessary diagnostic or therapeutic procedures, thereby negatively affecting patients' care (5). In this regard, the occurrence of a benign lesion mimicking malignancy was reported in a patient with fast-growing soft tissue nodules confined to the left groin. This was initially suspected as malignant due to conventional imaging features and an increased ^18^F-FDG uptake at PET/CT (Hu et al.). Unexpectedly, subsequent surgical excision revealed an uncommon localization of Kimura's disease, a rare benign chronic lympho-granulomatous disease. Based on the overall patient history, as well as clinical, laboratory, and imaging data, this case highlights the need to consider differential diagnoses for ^18^F-FDG-avid lymph nodes, ranging from malignant to inflammatory or infectious diseases, such as lymphoma, nodal metastases, active tuberculosis, sarcoidosis, or benign lympho-proliferative disorders, all of which can show a similar degree of tracer uptake (6). Similarly, Borè et al. reported a paramediastinal hypermetabolic lesion detected by ^18^F-FDG PET/CT, initially performed for staging in a patient with ovarian cancer and suspected for metastasis or synchronous primitive tumor. After a subsequent biopsy, mediastinal Schwannoma was instead diagnosed, allowing the patient to undergo chemotherapy and ovarian surgery, as initially planned. This case represents a warning for the risk of PET false positives that, particularly in oncological patients, may result in incorrect upstaging, in turn potentially leading to changes in the planned therapeutic management and consequently, exposing patients to overtreatment and associated toxicity risks. Conversely, the chance of misinterpreting a low ^18^F-FDG-avid tumor as a benign lesion requires careful consideration, with low metabolic activity in malignant lesions mainly related to low glucose metabolism (reflecting histologically and clinically less-aggressive tumor behavior), low cellularity, or small size (7). For example, oligo-vertebral plasma cell myeloma localizations, characterized by the coexistence of mild ^18^F-FDG uptake and morpho-structural changes, may overlap with the morpho-functional features of a vertebral hemangioma, potentially delaying tumor diagnosis and treatment (Hu et al.).

## Author contributions

ST, NQ, and GM drafted the manuscript and critically revised the final version. All authors gave their final approval for manuscript submission.

## Conflict of interest

The authors declare that the research was conducted in the absence of any commercial or financial relationships that could be construed as a potential conflict of interest.

## Publisher's note

All claims expressed in this article are solely those of the authors and do not necessarily represent those of their affiliated organizations, or those of the publisher, the editors and the reviewers. Any product that may be evaluated in this article, or claim that may be made by its manufacturer, is not guaranteed or endorsed by the publisher.
